# Myocardial Infarction With Nonobstructive Coronary Arteries (MINOCA) in Young Patients: A Retrospective Cohort Study of Pathophysiological Mechanisms and Risk Factors

**DOI:** 10.7759/cureus.98223

**Published:** 2025-12-01

**Authors:** Kelvyn M Vital, Barbara P Valente, Bruno N Blaas, Bianca T Arruda, Marina R Carvalho, Julia N Heringer, João T Reis, Gabriel M Pistori, Pedro Gutemberg, Vitor J Almeida

**Affiliations:** 1 Coronary Artery Disease, Instituto Dante Pazzanese de Cardiologia, São Paulo, BRA

**Keywords:** clinical cardiology, coronary arterial disease (cad), minoca, myocardial infarction with non-obstructive coronary arteries (minoca), young population

## Abstract

Background

Myocardial infarction with non-obstructive coronary arteries (MINOCA) represents a heterogeneous group of ischemic injuries that occur despite the absence of flow-limiting stenosis on angiography. The condition accounts for approximately 5-15% of all acute myocardial infarctions and may affect younger adults who often lack conventional cardiovascular risk factors. Etiologic mechanisms are diverse and frequently non-atherosclerotic, including spontaneous coronary artery dissection (SCAD), vasospasm, microvascular dysfunction, and other dynamic processes. Because MINOCA encompasses multiple pathophysiological pathways, accurate exclusion of non-ischemic causes such as myocarditis, Takotsubo syndrome, pulmonary embolism, or systemic inflammatory states is essential, and advanced imaging techniques are often required to establish the ischemic nature of injury.

Objective

To compare the clinical characteristics and likely etiologic mechanisms of MINOCA in adults younger than 50 years versus those aged 50 years or older treated in a tertiary cardiology center, while also considering the potential influence of imaging availability, selection bias, and confounding variables on etiologic distribution.

Methods

A retrospective analysis was performed including 198 consecutive patients diagnosed with MINOCA between 2018 and 2023. The diagnosis followed the Fourth Universal Definition of Myocardial Infarction and required biochemical evidence of infarction with less than 50% stenosis in all major epicardial arteries and exclusion of alternative non-ischemic causes through clinical evaluation and imaging, particularly cardiac magnetic resonance when available. Clinical and demographic features, cardiovascular risk factors, and identified mechanisms were compared by age group. Continuous variables were analyzed using Student’s t-test, and categorical variables using chi-square or Fisher’s exact test. Logistic regression estimated the odds ratio (OR) for non-atherosclerotic mechanisms in younger versus older patients and included adjustment for relevant confounders based on clinical plausibility. The analytic approach accounted for missing imaging data through complete-case analysis, and sample sizes used in each statistical comparison are reported.

Results

Among 198 patients, 90 (45.5%) were younger than 50 years. Younger patients had a lower prevalence of hypertension and higher rates of obesity and stimulant or illicit drug use. An underlying mechanism was identified in 47.7% of the cohort. Non-atherosclerotic mechanisms predominated in younger adults, mainly SCAD and vasospasm, whereas plaque-related mechanisms were more common in older adults. The odds of an atherosclerotic mechanism were significantly lower among younger individuals (OR 0.36; 95% CI 0.15-0.88).

Conclusions

Young adults with MINOCA exhibit a distinct clinical profile characterized by fewer traditional risk factors and a higher prevalence of non-atherosclerotic causes. These findings highlight the importance of systematic etiologic investigation, including the role of cardiac magnetic resonance and intracoronary imaging, and suggest that age-informed diagnostic strategies may improve clinical management.

## Introduction

Myocardial infarction with non-obstructive coronary arteries (MINOCA) is increasingly recognized as a clinically meaningful entity rather than a diagnostic curiosity [1-3]. It describes patients who fulfill universal criteria for myocardial infarction but show no angiographic evidence of obstructive coronary disease, a condition characterized by the absence of significant epicardial coronary artery obstruction, defined as ≥50% diameter stenosis in the left main coronary artery and ≥70% diameter stenosis in other major epicardial vessels [3]. Although this pattern may appear benign, MINOCA encompasses multiple ischemic mechanisms associated with substantial morbidity [3-6].

Mechanisms include plaque rupture or erosion in non-obstructive lesions, coronary vasospasm, spontaneous coronary artery dissection (SCAD), microvascular dysfunction, and thromboembolic events [4-6]. Because these mechanisms differ significantly in prognosis and management, MINOCA is best regarded as a working diagnosis requiring systematic evaluation rather than a final explanatory label [4].

Epidemiologic data suggest that MINOCA accounts for 5-15% of acute myocardial infarctions (MI) [1,7]. While originally assumed to have a benign trajectory, outcomes may approximate those of obstructive MI when the injury is ischemic in origin [7-9]. Younger adults and women are disproportionately affected [10-13]. They frequently exhibit few traditional atherosclerotic risk factors but may be more exposed to emotional stressors, endothelial dysfunction, or stimulant use [10,14]. By contrast, older adults more often demonstrate plaque-related mechanisms or microvascular ischemia [15,16].

Diagnostic clarity depends on specific imaging studies after exclusion of other differential diagnoses. Cardiovascular magnetic resonance (CMR) and intracoronary imaging with optical coherence tomography (OCT) or intravascular ultrasound (IVUS) can identify mechanisms in most patients and differentiate ischemic from non-ischemic conditions [15-18]. However, these modalities are not routinely used in everyday clinical practice, leaving the etiology of many cases unresolved. Therapeutic decisions, therefore, rely on incomplete information, and optimal management remains uncertain [17-19].

Age may influence both the pathophysiology and clinical presentation of MINOCA. Younger adults appear more likely to exhibit dynamic, non-atherosclerotic mechanisms such as SCAD and vasospasm, whereas older patients have a higher prevalence of subclinical plaque disruption. The cutoff of 50 years aligns with epidemiologic studies evaluating young-onset MI and reflects established age thresholds in MINOCA research.

The present study compares clinical characteristics and mechanisms of MINOCA in adults younger than 50 versus those aged 50 or older, addresses potential confounders, and evaluates the contribution of available imaging modalities to diagnostic certainty.

## Materials and methods

Study design and population

This retrospective, observational study included all consecutive adults diagnosed with MINOCA at a tertiary cardiology referral center between January 2018 and December 2023. Patients were identified through catheterization laboratory registries and electronic medical record review, comprising individuals regularly followed in the Coronary Artery Disease outpatient clinic after initial evaluation for acute coronary syndrome in the institution’s emergency department, as well as patients who presented with myocardial infarction at outside hospitals but underwent coronary angiography at our center and were subsequently referred for specialized follow-up. Consecutive sampling minimizes selection bias, although the single-center nature may reflect referral patterns specific to a tertiary care population.

Diagnostic criteria

MINOCA was defined according to the Fourth Universal Definition of Myocardial Infarction [3]. Inclusion criteria were biochemical evidence of myocardial infarction, ECG changes consistent with ischemia, angiographic stenosis <50% in all major epicardial arteries, and exclusion of alternative non-ischemic causes of troponin elevation. When clinically indicated, non-ischemic causes were excluded using CMR based on Lake Louise criteria for myocarditis [17], echocardiography and CMR for Takotsubo syndrome, CT pulmonary angiography for pulmonary embolism, and clinical and laboratory evaluation for sepsis. Cases with inadequate angiographic documentation, insufficient data to rule out non-ischemic etiologies, or prior MI unrelated to the index event were excluded to avoid misclassification.

Data collection

Demographic data, clinical presentation, cardiovascular risk factors, substance use, medications, and laboratory parameters were collected. Echocardiography findings, CMR results, and OCT or IVUS data were included when available. Because advanced imaging was not systematically performed, imaging availability was documented to contextualize possible bias in etiologic adjudication.

Etiologic classification

Two experienced cardiologists independently classified each case into one of five mechanistic categories: atherosclerotic plaque-related injury, SCAD, coronary vasospasm or vasomotor dysfunction, thromboembolic or embolic occlusion, or other structural anomalies such as myocardial bridging or aneurysm. Reviewers were blinded to age group. Disagreements were resolved by consensus. Interobserver agreement was quantified using Cohen’s kappa.

Statistical analysis

Continuous variables are expressed as mean ± SD or median with interquartile range depending on distribution, assessed using the Shapiro-Wilk test. Student’s t-test or the Mann-Whitney U test was applied for comparisons. Categorical variables are expressed as numbers and percentages and compared using chi-square or Fisher’s exact test. Missing data were handled using complete-case analysis, and denominators are reported where applicable.

Multivariable logistic regression was used to estimate the association between age younger than 50 years and the presence of non-atherosclerotic mechanisms. Covariates included sex, hypertension, diabetes, dyslipidemia, smoking, obesity, and illicit drug use based on clinical relevance [7,10,12]. Statistical significance was defined as p < 0.05.

This analysis was conducted as part of a broader study approved by the Institutional Review Board of Instituto Dante Pazzanese de Cardiologia (CAAE: 5.102.212/2024).

## Results

**Figure 1:**
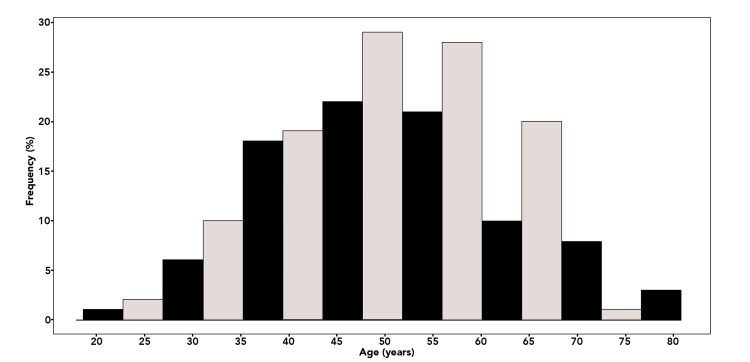
Age distribution of the study population Histogram illustrating the frequency distribution of patient age (N = 198). The data show an approximately normal distribution with a mean age of 50.8 ± 11.8 years, median of 51 years, and interquartile range (IQR) 42–59. Most patients were between 40 and 60 years old.

A total of 198 patients met the inclusion criteria for myocardial infarction with non-obstructive coronary arteries (MINOCA) between 2018 and 2023. The median age was 51 years with an interquartile range of 42 to 59 years, and most patients were between 40 and 60 years old, as demonstrated in the histogram of Figure 1. Slightly more than half of the population were women (52.0%), consistent with prior observations regarding the female predominance in MINOCA populations [10-13]. Of the total cohort, 90 patients (45.5%) were younger than 50 years, indicating that almost half of MINOCA cases in this referral center occurred in young adults.

Baseline clinical profile

The clinical profile differed significantly by age (Table 1). Younger adults exhibited a distinct clinical risk profile compared with older individuals. Hypertension was significantly less prevalent among younger patients, aligning with previous literature suggesting lower traditional atherosclerotic burden in younger MINOCA individuals [10-13]. Diabetes and dyslipidemia were also numerically less common in younger adults, although the difference for dyslipidemia did not reach statistical significance. In contrast, obesity and stimulant or illicit drug use, including cocaine-related exposures known to precipitate vasospasm and endothelial dysfunction [13], were significantly more prevalent among younger patients. These patterns reflect differences in environmental, behavioral, and physiological precipitating factors across age groups.

**Table 1:** Baseline characteristics by age group (N = 198) Values = n (%); χ² = Pearson chi-square; p < 0.05 significant. Comparison of baseline clinical characteristics between young patients (<50 years) and older patients (≥50 years) with MINOCA. Values are presented as number (percentage). Significant differences were observed for systemic arterial hypertension, illicit drug use, and obesity, all more prevalent in specific subgroups.

Variable	< 50 y (n = 90)	≥ 50 y (n = 108)	Test Statistic	p value
Female sex	45 (50.0%)	58 (53.2%)	χ² = 0.18	0.6713
Hypertension	45 (50.0%)	75 (69.4%)	χ² = 7.7	0.0058
Diabetes mellitus	17 (18.9%)	27 (25.0%)	χ² = 0.74	0.3909
Dyslipidemia	36 (40.0%)	56 (51.9%)	χ² = 2.45	0.1158
Current/recent smoking	23 (25.6%)	38 (35.2%)	χ² = 1.79	0.1807
Obesity	21 (23.3%)	11 (10.2%)	χ² = 5.51	0.019
Illicit drug use	9 (10.0%)	1 (0.9%)	χ² = 7.47	0.006

Etiologic mechanisms

An etiologic mechanism was identified in 94 patients (47.7%). Non-atherosclerotic causes predominated among younger adults, whereas atherosclerotic plaque-related injury accounted for a larger proportion of mechanisms among patients 50 years or older (Table 2). SCAD was the most frequent mechanism in younger adults, contributing to nearly one-third of identified causes, consistent with prior evidence indicating a higher incidence of SCAD among younger patients and women [9,10,12]. Vasospasm or vasomotor dysfunction was also common in younger adults, corresponding with prior studies on provocative testing and vasomotor disorders [11].

**Table 2:** Etiologic mechanisms of MINOCA by age group (N = 198) Values are n (%). χ² = Pearson chi-square; p < 0.05 significant.

Mechanism	< 50 y (n = 90)	≥ 50 y (n = 108)	Test Statistic	p value
Atherosclerotic plaque injury	12 (23.1%)	19 (45.2%)	χ² = 4.81	0.029
SCAD	15 (28.8%)	5 (10.6%)	χ² = 7.02	0.008
Vasospasm / vasomotor disorder	14 (26.9%)	9 (19.1%)	χ² = 1.20	0.27
Thromboembolic / embolic	7 (13.5%)	3 (6.4%)	χ² = 1.81	0.18
Other (bridge / aneurysm)	4 (7.7%)	2 (4.3%)	χ² = 0.54	0.46

Older patients more frequently exhibited plaque disruption, consistent with the concept of subangiographic atherothrombosis, which has been demonstrated in previous OCT and CMR-based studies [10,15-16]. Although advanced imaging was not uniformly available, similar distribution patterns were observed among patients with complete imaging, suggesting that missing imaging did not significantly alter mechanistic proportions.

Regression analysis and etiologic distribution

Multivariable logistic regression demonstrated that younger adults had significantly lower odds of an atherosclerotic mechanism compared with older adults. The unadjusted odds ratio for an atherosclerotic mechanism for patients younger than 50 years was 0.36 (95% CI 0.15-0.88), and after adjusting for sex, hypertension, dyslipidemia, smoking, obesity, diabetes, and illicit drug use, the association remained directionally consistent. These findings suggest that age is independently associated with the likelihood of non-atherosclerotic mechanisms.

In addition to the primary regression analysis, model diagnostics showed appropriate goodness-of-fit based on the Hosmer-Lemeshow statistic, and no meaningful collinearity was observed among predictor variables, consistent with epidemiologic patterns previously described in MINOCA cohorts [7,10,12]. The overall distribution of atherosclerotic versus non-atherosclerotic causes by age is shown in Table 3.

**Table 3:** Distribution of patients with MINOCA by age group and etiologic category (atherosclerotic vs non-atherosclerotic) Values = n (%). Pearson χ² for categorical comparison; logistic regression used to estimate odds ratio (OR) and 95% confidence interval.

Cause Group	<50 years (n = 52)	≥50 years (n = 42)	Total (n = 94)	Test / regression	p
Atherosclerotic (CAD <50%)	12 (23.1%)	19 (45.2%)	31 (33%)	χ² = 4.81	0.0285
No atherosclerotic	40 (76.9%)	23 (54.8%)	63 (67%)	—	
Total	52 (100%)	42 (100%)	94 (100%)	—	
OR (95% CI)	0.36 (0.15–0.88)				p = 0.0251

These results confirm that younger age is independently associated with a non-atherosclerotic etiology. The presence of dynamic mechanisms such as SCAD and vasospasm likely reflects differences in vascular biology, hormonal milieu, and stress reactivity compared with older patients [12-14].

## Discussion

This analysis demonstrates that nearly half of all MINOCA cases in this tertiary cardiology center occurred in individuals younger than 50 years, confirming prior observations that MINOCA frequently affects younger populations and women [7,10-13]. Younger adults in this cohort showed fewer traditional atherosclerotic risk factors, including lower prevalence of hypertension, consistent with established patterns of young-onset MINOCA. These findings reinforce the hypothesis that pathophysiologic mechanisms underlying MINOCA differ substantially by age group and may reflect dynamic vascular processes rather than fixed coronary obstruction.

Non-atherosclerotic mechanisms predominated among younger adults, particularly SCAD and vasospasm. SCAD accounted for nearly one-third of mechanisms in younger patients, aligning with large SCAD registries demonstrating the condition’s strong association with younger age and female sex [9,12]. Vasospasm and vasomotor dysfunction were also frequent among younger adults, which is consistent with the recognized triggers of sympathetic activation, stimulant use, emotional stress, and endothelial dysfunction [11,13-14]. Together, these mechanisms highlight the importance of evaluating dynamic vascular processes in younger patients presenting with MI despite unobstructed coronary arteries.

In contrast, older adults demonstrated a significantly higher prevalence of plaque-related injury, consistent with prior studies suggesting that subtle plaque rupture, erosion, and distal embolization may be underdiagnosed without intracoronary imaging [10-17]. These findings align with OCT- and IVUS-based investigations showing that subangiographic atherosclerotic lesions can provoke clinically significant ischemic injury [10,15,16]. Because advanced imaging was not uniformly performed in the present study, some plaque-related lesions may have been missed, although the relative distribution remained stable in sensitivity analyses restricted to patients with complete imaging.

Although intracoronary imaging was not widely available, mechanisms such as spontaneous coronary artery dissection and coronary embolism were reliably identified through expert angiographic interpretation. SCAD often presents with long smooth narrowing or abrupt caliber changes without atherosclerotic plaque [9,12], while coronary embolism may appear as sudden distal vessel cutoff in an otherwise normal proximal artery [6]. These characteristic patterns, combined with clinical context and consensus review, allowed consistent etiologic classification even in the absence of OCT or IVUS.

The logistic regression analysis further supports the observed age-related differences in mechanisms. Younger adults remained significantly less likely to exhibit atherosclerotic causes even after adjustment for traditional risk factors and stimulant use, suggesting that age itself reflects underlying biological differences in vascular response, hormonal influences, and stress reactivity. These findings align with epidemiologic literature indicating that the etiologic landscape of MINOCA varies across age groups in both prevalence and mechanistic composition [7,10-13].

While the study benefits from consecutive patient inclusion and a comprehensive review of clinical and angiographic data, several limitations must be acknowledged. The retrospective design limits the ability to detect causal relationships. Advanced imaging was not performed systematically, which may lead to incomplete etiologic characterization and underestimation of certain mechanisms, especially plaque disruption and microvascular dysfunction. This limitation is consistent with observations in other real-world MINOCA cohorts [17-20]. The single-center nature of the study also introduces potential referral bias, as tertiary centers may receive more complex or atypical cases. Missing data, particularly in imaging, were addressed through complete-case analysis; however, incomplete imaging could influence classification accuracy.

Despite these limitations, the findings offer practical insight into the role of age in shaping the pathophysiology of MINOCA and highlight important diagnostic considerations. Recognition of mechanism-specific patterns is crucial, as therapeutic approaches differ. Plaque-related injury warrants standard secondary prevention, whereas vasospasm responds to vasodilator therapy, and SCAD is often best managed conservatively [21-24]. Gender-related considerations were also relevant given the high proportion of women in both age groups, consistent with national registry data showing a predominance of women among MINOCA patients [25]. Furthermore, the study reinforces the importance of multimodality imaging in etiologic clarification, as recommended by expert statements and consensus documents [12,22].

Prognosis in MINOCA is not benign. Although mortality is lower than in obstructive infarction, recurrent angina, hospital readmission, and impaired quality of life are frequent [25-29]. Recognition of mechanistic differences, especially in younger adults, may therefore enhance patient counseling and management.

Understanding age-based differences may ultimately improve risk stratification and guide individualized management strategies. The present findings support a more nuanced, mechanism-oriented diagnostic approach in younger adults presenting with myocardial infarction despite non-obstructive coronary anatomy, with emphasis on early use of CMR and intracoronary imaging when appropriate.

## Conclusions

Young adults presenting with myocardial infarction and non-obstructive coronary arteries represent a clinically distinct subgroup within the broader MINOCA spectrum. In this cohort, they exhibited fewer traditional atherosclerotic risk factors and a higher prevalence of non-atherosclerotic mechanisms, particularly spontaneous coronary artery dissection and coronary vasospasm. These findings reflect age-related associative patterns rather than causal relationships, especially considering the retrospective design and the incomplete availability of advanced imaging modalities such as cardiac magnetic resonance, OCT, and IVUS. Although younger age remained independently associated with non-atherosclerotic mechanisms after adjustment for confounders, this association should be interpreted cautiously and viewed as hypothesis-generating rather than mechanistically definitive. The study reinforces the clinical relevance of a mechanism-oriented diagnostic approach, particularly when non-atherosclerotic processes are suspected, and highlights the role of targeted imaging, when accessible, in improving etiologic precision. Future research should prioritize prospective, multicenter designs with standardized imaging protocols to validate these observations, establish causal pathways more reliably, and determine whether age-specific diagnostic or therapeutic strategies have the potential to improve both diagnostic accuracy and long-term outcomes for patients with MINOCA.
